# Effect of In Vitro Gastrointestinal Digestion and Colonic Fermentation on the Stability of Polyphenols in Pistachio (*Pistacia Vera* L.)

**DOI:** 10.3390/ijms24054975

**Published:** 2023-03-04

**Authors:** Isabel Velasco-Ruiz, Elsy De Santiago, José Luis Ordóñez-Díaz, Gema Pereira-Caro, José Manuel Moreno-Rojas

**Affiliations:** 1 Department of Agroindustry and Food Quality, Andalusian Institute of Agricultural and Fisheries Research and Training (IFAPA), Alameda del Obispo, Avda. Menéndez-Pidal, s/n., 14004 Córdoba, Spain; 2 Departamento de Bromatología y Tecnología de los Alimentos, Campus Rabanales, Ed. Darwin-Anexo, Universidad de Córdoba, 14014 Córdoba, Spain; 3 Foods for Health Group, Instituto Maimónides de Investigación Biomédica de Córdoba (IMIBIC), 14004 Córdoba, Spain

**Keywords:** pistachio, (poly)phenols, in-vitro gastrointestinal digestion, bioaccessibility, colonic fermentation, human microbial metabolism

## Abstract

The aim of this study was to evaluate the impact of in vitro gastrointestinal digestion and colonic fermentation on the polyphenol compounds from different varieties of pistachio by UHPLC-HRMS analysis. The total polyphenol content decreased significantly, mostly during oral (recoveries of 27 to 50%) and gastric digestion (recoveries of 10 to 18%), with no significant changes after the intestinal phase. After in vitro digestion, the hydroxybenzoic acids and the flavan-3-ols were the main compounds found in pistachio, with respective total polyphenol contents of 73 to 78% and 6 to 11%. More specifically, the main compounds determined after in vitro digestion were 3,4,5-trihydroxybenzoic acid, vanillic hexoside and epigallocatechin gallate. The colonic fermentation affected the total phenolic content of the six varieties studied, with a recovery range of 11 to 25% after 24 h of fecal incubation. A total of twelve catabolites were identified after fecal fermentation, the main compounds being the 3-(3′-hydroxyphenyl)propanoic, 3-(4′-hydroxyphenyl)propanoic, 3-(3′,4′-dihydroxyphenyl)propanoic, 3-hydroxyphenylacetic acids and 3,4-dihydroxyphenyl-ɣ-valerolactone. Based on these data, a catabolic pathway for colonic microbial degradation of phenolic compounds is proposed. The catabolites identified at the end of the process are potentially responsible for the health properties attributed to pistachio consumption.

## 1. Introduction

Pistachio (*Pistacia vera* L.) has been one of the most valuable crops in recent years worldwide. This nut belongs to the Anacardiaceae family, and while it originates from the Middle East, due to the numerous health properties attributed to its consumption and its versatility for cultivation, it is now widespread in many regions.

A total of 1,205,532 tons of this valued crop were produced in 2020, the main world producers being United States, Turkey and Iran, while Greece, Spain and Italy are the main European producers [1]. In 2020, Andalusia was the second largest pistachio producer in Spain, representing 8% of the total area cultivated and 16% of the national production. This Spanish region demonstrated better adaptation of crops and farm practices, with pistachio becoming an emerging alternative in the nut sector in Andalusia [2].

Pistachios are one of the most popular and commercially valuable edible nuts in the world because of their traditional use dating back to prehistoric times [3]. Recently, pistachios have been recognized for their nutritional quality and their potential contribution to good health, proving to have a high antioxidant activity, cellular antioxidant activity and antiproliferative properties [4]. The most important bioactives in pistachios are polyphenols, carotenoids, tocopherols and unsaturated fatty acids, which have led to numerous studies on the potential health properties of pistachios [5]. Specifically, it has been shown that polyphenols could prevent the formation of pro-oxidants by blocking the action of reactive oxygen species, thus playing an important role in the positive cardioprotective, anti-diabetic and anti-inflammatory effects on health [6]. Clinical studies have reported that pistachio consumption has a positive influence on oxidation biomarkers and antioxidant defenses. Hydrophilic phenol-rich extracts from both the skin and kernel of pistachio have been shown to inhibit the expression of proinflammatory markers and significantly decrease the total oxidant status in healthy subjects [7]. Describing the antioxidant and disease-preventive properties of pistachios would be interesting to increase their value for the industry.

In order to better understand the physiological response to polyphenols present in pistachios, it is important to know how pistachio polyphenols are absorbed and metabolized through the gastrointestinal tract. In fact, following ingestion, the release of the aglycone form of polyphenols occurs in the upper gastrointestinal tract and further undergoes phase II metabolism in epithelial/hepatic cells, appearing in the systemic circulation as the sulfate, glucuronide and methylated derivatives [8]. However, only a small fraction of the ingested polyphenols is fully absorbed in the upper GI tract. A substantial amount of the ingested polyphenols pass from the small to the large intestine where they undergo catabolic processes by the gut microbiota and are transformed into smaller phenolic compounds which can be further metabolized by phase II conjugation reactions occurring in the colonocytes or in the liver [9], therefore impacting human health [10]. Therefore, the biological activities of phenolic compounds may be mediated by their metabolites and catabolites, so it is crucial to elucidate the impact of the gastrointestinal digestion and colonic fermentation of polyphenols in dietary foods. To this end, in vitro methods have been widely used to efficiently simulate digestion processes and to study metabolization phenomena at different stages of digestion [11,12,13].

Despite significant advances in the last decade, to date, comprehensive data on the transformation of pistachio polyphenols by the gastrointestinal tract and the gut microbiota is still scarce [14,15,16,17]. There are two reports that evaluated the bioaccessibility of pistachio polyphenols after in vitro gastrointestinal digestion [15,16], without taking into consideration the large intestine. More recently, Rocchetti et al. [18] evaluated the bioaccessibility of polyphenols after in vitro GI digestion and colonic fermentation of edible nuts, including pistachio. However, this study used pig feces inoculum in the fecal fermentation, instead of human feces, and made a semi-quantification of the polyphenols. Herbello-Hermelo et al. [19] also evaluated the bioaccessibility of nuts and seeds, including pistachio, by an in vitro dialyzability approach, but focused only on the total polyphenol content, without showing the changes in the individual polyphenol profiles. Moreover, as far as we know, there is no report on the evaluation of the bioavailability of pistachio polyphenols in humans. Accordingly, further studies are needed to fully elucidate the transformation during gastrointestinal digestion and colonic fermentation of pistachio polyphenols. Furthermore, the use of additional pistachio varieties with different polyphenol profiles and concentrations (in total and relative content) would be desirable to further explore the influence of the digestion and fermentation process on pistachio polyphenols, with great interest in understanding their transformation and how this could affect their potential health benefits. Therefore, the aim of this study was to evaluate the impact of an in vitro digestion and colonic fermentation process on the polyphenol profiles of six pistachio varieties.

## 2. Results and Discussion

### 2.1. Changes in Polyphenolic Contents of Pistachio after Simulated Gastrointestinal Digestion and Bioaccessibility

A total of 58 polyphenol compounds, belonging to the different families, were identified in the pistachios samples before simulated gastrointestinal digestion. The HPLC-HRMS characteristics of the polyphenols identified in the pistachio varieties are shown in Table S1. The polyphenols identified in the pistachio samples included fifteen hydroxybenzoic acids, ten galloyl derivates, seven hydroxycinnamic acids, three flavones, eight flavonols, nine flavan-3-ols, four flavanones, one flavanonol and one stilbene. Among them, the group of hydroxybenzoic acid derivatives, including 3,4-dihydroxybenzoic acid, vanillic acid hexoside, benzoic acid derivate II and 3,4,5-trihydroxybenzoic acid, were the main polyphenols present in the pistachio samples, representing 97.7 to 98.5% (Table 1), followed by the flavan-3-ols, such as epigallocatechin gallate, catechin, epicatechin and gallocatechin, which ranged from 88.3 to 97.6% depending on the pistachio variety (Table 1). Remarkably, all families of polyphenols shared the same behavior when undergoing gastrointestinal digestion.

Overall, before in vitro oral digestion (BOD), Aegina (21.9 nmol/g DW) and Larnaka (18.2 nmol/g DW) presented the highest total polyphenol content, followed by the Sirora (15.5 nmol/g DW) and Kastel (11.1 nmol/g DW) varieties. The pistachio varieties showing the lowest polyphenol content were Golden Hills (7.4 nmol/g DW) and Kerman (7.0 nmol/g DW).

After in vitro oral digestion (AOD), the concentration of total phenolic compounds of the pistachio significantly decreased in all varieties, with recoveries ranging from 27 to 50% (Table 1). The Aegina, Golden Hills, Kastel and Sirora varieties were particularly affected after oral digestion, with decreases in their total polyphenol content ranging from 70 to 73%. After gastrointestinal digestion (AGD), the concentration of total phenolic compounds significantly decreased in all the varieties, with recovery rates from 10 to 18% (Table 1), while intestinal digestion (AID) did not show a significant impact on the total concentration of polyphenols, except for the Kerman variety, which presented a significant decrease in the amount of polyphenols (Table 1). These results are in keeping with Di Lorenzo et al. (2021) [6] and Mandalari et al. (2013) [15], who showed that pistachio polyphenols are mainly released during the digestive phase of digestion, with little or no release during the intestinal phase. It is noteworthy that the different families of polyphenols, including the hydroxybenzoic acids and the flavan-3-ols, are significantly reduced after oral and gastric digestion, with almost no effect by the intestinal digestion in all pistachio varieties (Table 1).

More specifically, among the individual phenolic compounds, 3,4-dihydroxybenzoic acid and vanillic acid hexoside were the main polyphenols determined in the pistachio varieties. Their trend was similar in all the varieties. In the case of 3,4-dihydroxybenzoic acid, it decreased significantly after gastric digestion, with very low recoveries, from 1 to 4%. For vanillic acid hexoside, the effect of gastric digestion was also important, but with recoveries of 54 to 80%. The intestinal phase did not result in a significant decrease in both compounds for all the varieties (Table 2). Other compounds, including 3,4,5-trihydroxybenzoic acid, pyrogallol, galloylshikimic acid, ellagic acid and benzoic acid derivate I, although found in trace quantities in pistachio varieties, showed a different behavior during the gastrointestinal digestion. Indeed, there was a significant increase after oral digestion of these compounds in all the pistachio varieties except for galloylshikimic acid, which even decreased in the Golden Hills, Kastel and Kerman varieties, with no increase in the other varieties. During the gastric phase, levels of 3,4,5-trihydroxybenzoic acid, galloylshikimic acid and ellagic acid only decreased in the Larnaka variety. Pyrogallol and benzoic acid derivate I decreased in all the varieties. In the intestinal phase, the content of all the compounds decreased in all the varieties, except for ellagic acid, which did not decrease in any variety (Table 2).

Regarding the second main group of polyphenol compounds in pistachio, Table 3 shows a significant decrease in the total flavan-3-ol content in all varieties and for all identified compounds. More specifically, epigallocatechin gallate, followed by gallocatechin and catechin, the main flavan-3-ols present in all pistachio varieties, showed a decrease in their concentration after oral digestion, this being significant in the Aegina, Kastel, Kerman and Larnaka varieties. This decrease after oral digestion was also significant for Golden Hills and Sirora in the case of catechin and epigallocatechin content. During the gastric phase, these three compounds decreased significantly in all the varieties, while in the intestinal phase, this decrease was only significant in the Larnaka variety for catechin and in the Kerman variety for epigallocatechin (Table 3).

### 2.2. Evolution of Phenolic Compounds of Pistachios during Colonic Fermentation

**Figure 1:**
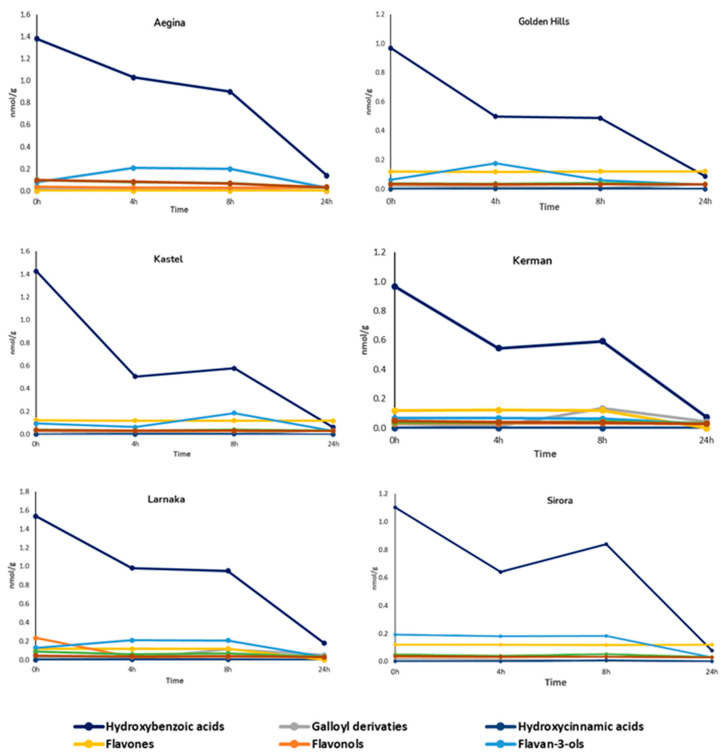
Degradation profiles of polyphenols in pistachio varieties during 24 h of in vitro colonic fermentation.

The digested pistachio samples were incubated with human feces for up to 24 h and analyzed by UHPLC-HRMS. The time-course profiles of the degradation of different groups of polyphenols from the different pistachio varieties are presented in Figure 1. In general, the colonic fermentation influenced the total phenolic content of the six varieties of pistachios, with respective recovery ranges of 10.8 and 24.6% for Sirora and Golden Hills varieties after the 24 h of fecal incubation. At the beginning of the colonic fermentation, hydroxybenzoic acids were the main polyphenolic group in all the pistachio varieties, accounting for 71 and 80% of the total content. After 24 h, there was a significant decrease in all varieties (recoveries from 7% in the Sirora variety, to 15% in the Larnaka variety). The group of flavones was more stable throughout the fermentation process for all the varieties of pistachios. Hydroxycinnamic acids and stilbenes were completely metabolized within the 24 h by the intestinal microbiota.

At the end of the fecal colonic incubation (24 h), the main group of polyphenols was hydroxybenzoic acids in Aegina, Kerman and Larnaka, and flavones in the case of the Golden Hills, Kastel and Sirora varieties.

The individual phenolic compound profile of hydroxybenzoic acids is represented in Table S3, showing a decreasing trend for all the compounds, with complete degradation for compounds such as theogallin, galloylshikimic acid, vanillic acid hexoside, ellagic acid and benzoic acid II. Finally, after the 24 h of fecal incubation, only three compounds remained in all the varieties, namely 3,4-dihydroxybenzoic acid, the predominant compound, benzene-1,2-diol and 3,4,5-trihydroxybenzoic acid (Table S3).

Table S4 shows the individual phenolic compound profile of flavan-3-ols. Although the concentration of the flavan-3-ol group increased after 4 h and 8 h of the colonic fermentation in Aegina, Golden Hills, Kerman, Larnaka and Kastel, this group showed a significant decrease in all the varieties after 24 h. Individually, it can be observed that most of the compounds of this group were degraded by the colonic microbiota after 24 h, including catechin, epigallocatechin gallate, epiafzelechin3-gallate and afzelechin III (Table S4). Epicatechin and epicatechin gallate remained after 24 h of fecal fermentation.

Moreover, after 24 h of colonic fermentation, twelve catabolites were identified and quantified in the digested pistachio samples. The details of their identification are shown in Table S2. The quantities of these phenolic catabolites are shown in Table 4. These catabolites, corrected for the endogenous levels of compounds found in the fecal fermentation medium, are arguably obtained from the microbial degradation of the parent compound present in pistachio samples.

Among them, 3-(3′-hydroxyphenyl)propanoic acid is the main compound in all the varieties, ranging from 70% of the total catabolites in Kerman, to 37% of the total in the Sirora variety. The second main compound was 3-hydroxyphenylacetic acid for all the varieties except Aegina, ranging from 18 to 33% of the total content of catabolites identified. Other catabolites identified in significant amounts were 3-(4′-hydroxyphenyl)propanoic acid, which was the second main compound in the Aegina variety, 3-(3′,4′-dihydroxyphenyl)propanoic acid and 3,4-dihydroxyphenyl-gamma-valerolactone (Table 4). After 24 h of fermentation, other phenolic compounds, including 3-(phenyl)propanoic acid, 3′-hydroxy-4′-methoxycinnamic acid, 4-hydroxybenzoic acid, 3-(4′-hydroxy-3′-methoxyphenyl)propanoic acid, 3,4-dihydroxyphenylacetic acid, 3-phenylacetic acid and 4′-hydroxy-3′-methoxycinnamic acid, appeared in the fermentate in trace amounts (Table 4).

**Figure 2:**
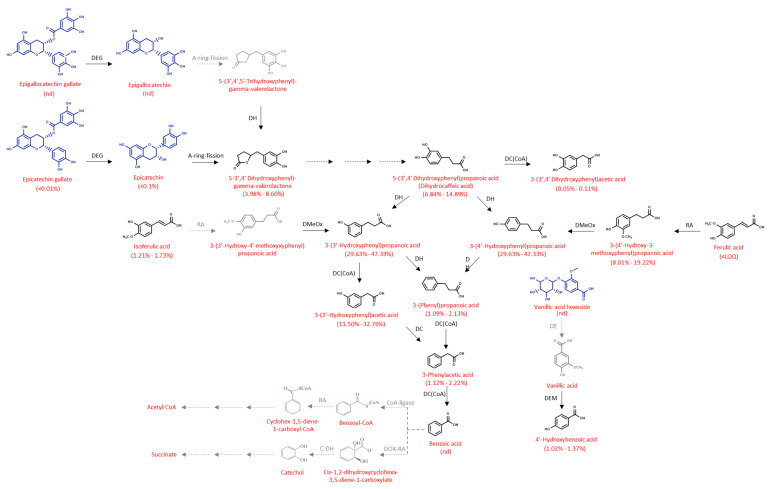
Tentative catabolic pathway for pistachio polyphenols by the colonic microbiota. Compounds in blue were presented before fecal fermentation, compounds in black were formed during fecal fermentation and compounds in dotted line were not detected. DEG: degalloylation; DES: de-esterification; DC: decarboxylation; RA: reductase; DMeOx: de-methoxyesterase; DH: dehydrogenase; DEM: demethylation; DOX-RA: dioxygenase reductase; C-DH: carboxylate dehydrogenase. The recovery ranges (%) of each compound are included for the six pistachio varieties after 24 h of in vitro fermentation.

The results enabled a catabolic pathway to be proposed for the formation of these phenolic catabolites during fecal fermentation (Figure 2). The colonic degradation could be primarily produced from phenolics present originally in pistachio, such as the flavan-3-ol group, including the epigallocatechin gallate and epicatechin gallate, leading to the formation of epigallocatechin and epicatechin via degalloylation, respectively. It is proposed that the microbial-mediated catabolism would begin opening the A-ring of epigallocatechin and epicatechin to the 5-(3′,4′,5′-trihydroxyphenyl)gamma-valerolactone, not identified in our study, and to 5-(3′,4′-dihydroxyphenyl)-gamma-valerolactone, respectively [20] (Figure 2). In addition, via dehydroxylation, the 5-(3′,4′,5′-trihydroxyphenyl)gamma-valerolactone would result in the formation of 5-(3′,4′-dihydroxyphenyl)-gamma-valerolactone. Moreover, this compound would be subject to dehydroxylation of the phenyl ring, and side-chain shortening would result in the formation of 3,-(3′,4′-dihydroxyphenyl)propanoic acid. This compound was further degraded to 3-(3′,4-dihydroxyphenyl)acetic acid by the action of the microbiota via decarboxylation, but also 3-(3′-hydroxyphenyl)propanoic acid and 3-(4′-hydroxyphenyl)propanoic acid by dehydroxylation. In addition, the microbiota degraded isoferulic acid to produce 3-(3′-hydroxy-4′-methoxyphenyl)propanoic acid, which could subsequently form 3-(3′-hydroxyphenyl)propanoic acid by a de-methoxylation step, mediated by the microbiota. Similarly, the action of the microbial-mediated reductase on ferulic acid could produce 3-(4′-hydroxy-3′-methoxyphenyl)propanoic acid and subsequently the 3-(4′-hydroxyphenyl)propanoic acid via demethoxylation.

After 8 h of colonic incubation, the catabolite 3-(3′-hydroxyphenyl)acetic acid was identified in the Golden Hills, Kerman and Sirora varieties, resulting from the transformation of 3-(3′-hydroxyphenyl)propanoic via decarboxylation (Figure 2). During the fecal fermentation process, this catabolite increased, being detected after 24 h in great amounts in all the pistachio varieties.

Moreover, the catabolite 3-(phenyl)propanoic acid, identified in the fecal fermentation of the pistachio varieties, would be formed from the 4′-dehydroxylation and 3′-dehydroxylation of the 3-(3′-hydroxyphenyl)propanoic acid and 3-(4′-hydroxyphenyl)propanoic acid, respectively (Figure 2). Additionally, the 3-(phenyl)propanoic acid would be subjected to subsequences decarboxylation steps to yield 3-phenylacetic acid and benzoic acid. The compound 3-(3′-hydroxyphenyl)acetic acid could also contribute to the amount of 3-phenylacetic acid in the fermentation via decaboxylation (Figure 2). Finally, benzoic acid would be degraded through different metabolic pathways as indicated in Figure 2 [21,22].

On the other hand, the hydroxybenzoic acid group, such as vanillic acid hexoside, one of the most abundant phenolic acids in pistachio samples, was transformed to vanillic acid via deglycosidation, and subsequently further degraded by microbial activity to 4-hydroxybenzoic acid via demethylation, as shown in Figure 2. At last, 4-hydroxybenzoic acid would be degraded as indicated in Figure 2 [21,22].

The results show a high bioconversion of polyphenolic compounds during fermentation, which agrees with results obtained in other studies on pistachios [17] and other nuts [18]. Nevertheless, literature related to the bioaccessibility of their catabolites after colonic fermentation on pistachios or other nuts is very limited. For instance, Rocchetti et al. [23] identified six catabolites in pistachios derived from polyphenolic compounds, finding 3-(3′,4′-dihydroxyphenyl)acetic acid to be the most abundant catabolite after 24 h of the colonic fermentation of pistachio. The phenolic acids 4-hydroxybenzoic acid, hippuric acid, 3,4-dihydroxycinnamic acid, 3,4-dihydroxybenzoic acid and 3,4-dihydroxybenzaldehyde, have been identified as degradation products of parent pistachio polyphenols by the colonic microbiota, 4-hydroxybenzoic acid, 3,4-dihydroxybenzaldehyde and 3,4-dihydroxyphenylacetic acid, being the most discriminant compounds of the changes in the phenolic profile observed during colonic fermentation [23]. These results are partially in keeping with the results obtained in our study, where we have additionally identified 3-hydroxyphenylacetic acid, 3-(3,4-dihydroxyphenyl)propanoic acid (dihydrocaffeic acid), 3-(4′-hydroxyphenyl)propanoic acid, 3-(3′-hydroxyphenyl)propanoic acid and 3,4-dihydroxyphenyl-ɣ-valerolactone as the major phenolic catabolites presented after the colonic fermentation of different pistachio varieties. Moreover, it was found that the catabolite 3-(3′-hydroxyphenyl)propanoic acid was one of the major products 5 h after incubation of hazelnut skin or hazelnut skin extract with human feces [24].

Our results are also in line with human bioavailability studies of almond, pecan and walnut polyphenols after consumption of whole nuts or crude extracts. For instance, 5-(3,4-dihydroxyphenyl)-ɣ-valerolactone, hydroxycinnamic, hydroxyphenylacetic and hydroxybenzoic acid catabolites were determined in urine after the consumption of almond skin extract [25]. Urinary 3-hydroxyphenylacetic acid, a microbial phenolic metabolite, also increased 13–24 h after pecan consumption [26]. Moreover, (hydroxy)phenyl-ɣ-valerolactones appeared in urine after hazelnut skin drink ingestion as predominant colonic catabolites of flavan-3-ol monomers in the hazelnut skin drink [27]. All these results highlight an extensive bioconversion of parent pistachio phenolic compounds due to colonic microbiota catabolism producing a wide range of phenolic metabolites, which could be exported into the systemic circulation.

## 3. Materials and Methods

### 3.1. Chemicals

Methanol (MeOH) HPLC grade, acetone and potassium hydroxide were acquired from Panreac Applichem ITW Reagents (Darmstadt, Germany). Sodium chloride and magnesium chloride hexahydrate were purchased from Fisher Scientific (Madrid, Spain); 6-hydroxy-2,5,7,8-tetramethylchroman-2-carboxylic acid (Trolox), 2,2′-azinobis-(3-ethylbenzothiazoline-6-sulphonic acid) diammonium salt (ABTS), sodium bicarbonate and ammonium carbonate were supplied by Sigma-Aldrich (Madrid, Spain); and potassium dihydrogen phosphate, sodium hydrogen carbonate, magnesium sulfate monohydrate and potassium hydrogen phosphate were obtained from VWR International Eurolab (Barcelona, Spain). Yeast extracts, peptone, tween 80, hemin, vitamin K, L-cysteine hydrochloride monohydrate, resazurin redox indicator and calcium chloride were acquired from Sigma-Aldrich (Madrid, Spain). α-Amylase from human saliva (300–1500 U/mg protein), pepsin (3.2–4.5 U/mg protein), pancreatin from porcine pancreas (4× UPS), bile salts and calcium chloride were purchased from Sigma-Aldrich (Madrid, Spain). Reference standard compounds, including 3,4,5-trihydroxybenzoic acid (gallic acid), benzene-1,2,3-triol (pyrogallol), benzoic acid, 4-hydroxybenzoic acid, 4-hydroxy-3-methoxybenzoic acid (vanillic acid), ellagic acid, sinapic acid, 4′-hydroxycinnamic acid, 4′-hydroxy-3′-methoxycinnamic acid, 3′-hydroxy-4′-methoxycinnamic acid, catechin, 3-(3,4-dihydroxycinnamoyl)quinic acid, epigallocatechin gallate, epicatechin gallate, naringenin, myricetin, quercetin 3-rutinoside, luteolin, quercetin, 3-(3′,4′-dihydroxyphenyl)propanoic acid, 3-(3′-methoxy,4′-hydroxyphenyl)propanoic acid, 3-phenylpropanoic acid, 3′,4′-dihydroxyphenylacetic acid, 3-phenylacetic acid and 4′-hydroxyphenylacetic acid, were purchased from Sigma-Aldrich (Madrid, Spain). The acetonitrile and methanol were of LC-MS grade.

### 3.2. Materials and Sample Preparation

Pistachios (*Pistacia vera* L.) were kindly provided by an experimental field of pistachio varieties of IFAPA in Guadix (Granada, Spain), which were planted in 2012. Six varieties were selected: Aegina, Golden Hills, Kastel, Kerman, Larnaka and Sirora. Fresh pistachios were peeled and ground using a homogenizer (SAMMIC, Madrid, Spain), and stored at −80 °C until analysis.

### 3.3. In Vitro Gastrointestinal Digestion

An in vitro gastrointestinal digestion procedure was performed according to Moreno-Ortega et al. [11]. Three steps simulated the oral, gastric and intestinal conditions, the details of which are described in Moreno-Ortega et al. [10]. The whole process took place in a stirred water bath (Unitronic Reciprocating Shaking Bath, model 6032011, J.P. Selecta, Barcelona, Spain) at 37 °C, with 100 mL amber glass bottles containing 2 g of each sample in triplicate. During the oral phase, 2 g of lyophilized sample, 14 mL of simulated salivary fluids containing 250 μL of α-amylase (1.3 mg/mL), 0.1 mL of 0.3 M CaCl_2_ and 5.65 mL of distilled water were shaken at 37 °C for 5 min. Then, the pH was adjusted to 3 using 1 M HCl. A total of 15 mL simulated gastric fluids was added to the samples, together with 1.19 mL of pepsin (0.1 g/mL), 0.01 mL of 0.3 M CaCl_2_ and 3.8 mL of distilled water. The mixture was incubated at 37 °C for 120 min. Finally, for the intestinal phase, 22 mL of the simulated intestinal fluids were added to the samples, together with 10 mL of pancreatin solution (8 mg/mL), 5 mL of bile salts (25 mg/mL), 0.08 mL of 0.3 M CaCl_2_ and 9.92 mL of distilled water. Then, 1 M NaOH solution was used to adjust the pH to 7. The mixture was incubated for 120 min at 37 °C. Samples were taken before (BOD) and after oral digestion (AOD), after gastric (AGD) and after intestinal digestion (AID) in individual experiments. These samples were lyophilized and stored at −80 °C. The bioaccessibility index was calculated as the compound concentration after simulated gastrointestinal digestion, divided by the compound concentration in non-digested samples.

### 3.4. In Vitro Colonic Fermentation

The freeze-dried digested pistachio samples were subjected individually to in vitro fermentation, to simulate the conditions in the colon following the method described by De Santiago et al. [28] and adapted to our laboratory, including the details of the composition of the growth medium. The human fecal samples were obtained from three healthy, non-smoking volunteers, who had not consumed antibiotics for at least 6 months prior to the study. The volunteers followed a low polyphenol diet for 48 h before the fecal sample collection. The samples were collected by the donors in plastic tubes, containing an AnaeroGen sachet (Oxoid Ltd., Cambridge, UK) to maintain anaerobic conditions during transportation, and were processed within 30 min of passage. A fecal slurry was prepared by homogenizing the feces in pre-reduced phosphate buffered saline (PBS). The temperature of the incubation was set to 37 °C using a Unitronic OR circulating water bath (JP Selecta, Abrera, Spain), and the fermentation bottles were inoculated with the fecal slurry (10% *w/v* of fresh human feces) for a period of 48 h. After the addition of the lyophilized digested pistachio samples (200 mg), the bottles were purged with oxygen free nitrogen (OFN) and were sealed airtight, and the anaerobic conditions were maintained by using a continuous OFN flow. Aliquots (1 mL) of fecal suspensions were taken after 0, 4, 8 and 24 h. The samples were centrifuged at 13,500 rpm at 4 °C for 10 min and stored immediately at −80 °C until analysis.

### 3.5. Extraction of Polyphenols

The extraction of polyphenols from the in-vitro-digested samples and the fecal samples was adapted from Ordóñez-Díaz et al. [12] with some modifications. For the in-vitro-digested samples, 0.2 g of lyophilized sample was homogenized with 1 mL of a methanol/acidified water mixture (80:20, v/v) with 0.1% formic acid. The samples were centrifuged at 5000 rpm for 10 min at 4 °C, and supernatants were collected. The pellet was reextracted with 1 mL of the same solvent, as described above. All the supernatants were pooled to a final volume of 2 mL. For the fermented pistachio samples, 0.5 mL of the fecal incubates were extracted using 0.5 mL of 0.1% formic acid in methanol/acidified water (80:20, *v*/*v*), vortexed and centrifuged at 5000 rpm for 10 min at 4 °C, and supernatants were collected.

### 3.6. UHPLC-HRMS Analysis

The identification and quantification of polyphenols in the pistachio samples were conducted using an UHPLC-HRMS mass spectrometer system (Thermo Scientific, San José, Ca. USA), comprising an UHPLC pump and an autosampler operating at 4 °C (ThermoFisher Scientific, San Jose, CA, USA). The phenolic compounds were separated on a Zorbax SB-C18 RRHD column (100 × 2.1 mm i.d., 1.8 µm (Agilent, Santa Clara, CA, USA)), preceded by a guard pre-column of the same stationary phase, and maintained at 40 °C. The flow rate was set to 0.2 mL/min, with a 26 min gradient of phase A: deionized water with 0.1% formic acid and B: acetonitrile with 0.1% formic acid. The gradient started at 3% B, was maintained for 2 min, then rose to 65% B in 18 min, before rising to 80% B in 1 min and being maintained for 6 min, for a total of a 26 min gradient. After that, the column was equilibrated to the previous conditions within 10 min. The eluate (0.2 mL/min) went into an Exactive Orbitrap mass spectrometer (Thermo Scientific, San José, CA, USA) fitted with a heated electrospray ionization probe (HESI), operating in a negative ionization mode, for the determination of polyphenols. Full scans were recorder in m/z range from 100 to 1200, with a resolution of 50,000 Hz and a full AGC target of 100,000 charges, using 2 microscans. Analyses were also based on scans with an in-source collision-induced dissociation (CID) at 25.0 eV. The capillary temperature of the MS experiment with HESI in the negative ionization mode was 320 ºC, the sheath gas was 35 units, the heater temperature was 150 ºC, the auxiliary gas was 10 units, and the spray voltage was 4.0 kV. Data acquisition and processing were carried out using the Xcalibur 3.0 software (Thermo Scientific, San José, CA, USA).

Targeted identification of phenolic compounds was achieved by comparing the exact mass and the retention time with available standards. In the absence of standards, compounds were tentatively identified by comparing the theoretical exact mass of the molecular ion with the measured accurate mass of the molecular ion and then searching metabolite databases, including Metlin, Phenol Explorer and more general chemical databases, such as PubChem and ChemSpider. Compounds having molecular masses within the pre-specified tolerance (≤10 ppm) of the query masses were retrieved from these databases. The phenolic compounds were quantified by selecting the theoretical exact mass of the molecular ion by reference to standard curves. In the absence of reference compounds, they were quantified by reference to the calibration curve of a closely related parent compound.

### 3.7. Statistical Analysis

Two-way ANOVA and Tukey’s post-hoc tests were applied to identify the differences among the samples, using the R software (v. 3.6.3, R Core Team, Vienna, Austria).

## 4. Conclusions

This study reports the stability and bioaccessibility of polyphenolic compounds in pistachio during in vitro digestion and simulated fecal fermentation. During the in vitro digestion process, a significant decrease in total polyphenol content occurred during the oral and gastric phases. In particular, significant decreases were observed in the two main groups of compounds: hydroxybenzoic acids and flavan-3-ols. The Kerman and Larnaka varieties showed the highest bioaccessibility after complete digestion, followed by Golden Hills and Sirora. Aegina was the variety with the lowest bioaccessibility of the compounds studied. The fecal fermentation process transformed the phenolic compound profile of pistachio into its degradation catabolites. After 24 h of incubation, the main groups of polyphenols were hydroxybenzoic acids for the Aegina, Kerman and Larnaka varieties, and flavones for the Golden Hills, Kastel and Sirora varieties. Moreover, twelve catabolites could be identified and quantified by UHPLC-HRMS for the six pistachio varieties analyzed. These results of the in vitro fecal fermentation allowed us to propose a route of degradation or transformation of the pistachio phenolic compounds by the intestinal microbiota. After 24 h fermentation, the five main catabolites identified were 3-(3′-hydroxyphenyl)propanoic acid, 3-(4′-hydroxyphenyl)propanoic acid, 3-hydroxyphenylacetic acid, 3-(3′,4′-dihydroxyphenyl)propanoic acid and 3,4-dihydroxyphenyl-gamma-valerolactone. These could be the major compounds at the end of the fermentation process and ones responsible for the possible health benefits associated with pistachio consumption.

## Figures and Tables

**Table 1:** Polyphenol families quantified by pistachio varieties before and after oral, gastric and intestinal in vitro digestion (nmol/g DW).

	Hydroxybenzoic Acids	Galloyl Derivatives	Hydroxycinnamic Acids	Flavones	Flavonols	Flavan-3-ols	Flavanones	Flavanols	Stilbenes	Total
**Aegina**										
BOD	18.2 a	0.1 a	0.01 b	0.08 b	0.09 a	3.1 a	0.2 a	0.02 c	0.1 b	21.9 a
AOD	4.3 b	0.08 b	0.03 a	0.03 c	0.05 b	1.6 b	0.12 b	0.02 cd	0.3 a	6.5 b
%	24	69	325	44	54	52	63	87	275	30
AGD	1.6 c	0.03 c	0.01 b	0.01 d	0.03 cd	0.4 c	0.12 bc	0.01 de	0.08 c	2.3 c
%	9	26	82	15	31	14	61	60	75	10
AID	0.9 c	0.02 cd	0.00 c	0.01 d	0.02 d	0.1 c	0.09 bcd	0.01 e	0.06 c	1.2 c
%	5	15	34	11	26	4	47	45	56	6
**Golden Hills**										
BOD	6.4 a	0.11 a	0.010 bc	0.011 a	0.044 a	0.57 a	0.03 a	0.017 a	0.04 b	7.3 a
AOD	1.3 b	0.10 a	0.070 a	0.008 b	0.028 b	0.31 b	0.023 b	0.016 a	0.1 a	2.0 b
%	21	90	729	70	63	54	81	93	268	27
AGD	0.6 b	0.02 b	0.021 b	0.009 ab	0.017 c	0.09 c	0.020 b	0.01 b	0.04 b	0.8 bc
%	9	16	219	81	38	16	72	54	103	11
AID	0. b	0.02 b	0.007 c	0.009 ab	0.013 c	0.04 c	0.020 b	0.005 b	0.03 b	0.6 c
%	7	20	76	79	30	6	72	33	86	8
**Kastel**										
BOD	9.6 a	0.14 a	0.01 c	0.05 a	0.06 a	1.1 a	0.03 a	0.017 a	0.06 b	11.1 a
AOD	2.2 b	0.11 b	0.10 a	0.02 b	0.03 b	0.6 b	0.017 b	0.017 a	0.14 a	3.2 b
%	23	76	990	47	56	52	60	95	237	29
AGD	0.8 bc	0.03 c	0.02 b	0.01 c	0.01 c	0.1 c	0.014 b	0.007 b	0.04 c	1.1 c
%	8	18	210	20	23	10	46	43	70	10
AID	0.5 c	0.01 d	0.01 c	0.01 c	0.01 c	0.04 c	0.012 b	0.005 c	0.04 c	0.6 c
%	5	7	76	18	17	4	41	28	64	5
**Kerman**										
BOD	5.5 a	0.2 a	0.09 b	0.05 a	0.07 a	1.00 a	0.04 a	0.03 b	0.06 b	7.0 a
AOD	2.4 b	0.1 a	0.040 a	0.02 b	0.05 b	0.60 b	0.03 ab	0.04 a	0.18 a	3.5 b
%	44	74	552	48	67	55	69	173	287	50
AGD	0.9 c	0.04 b	0.01 b	0.01 b	0.02 c	0.16 c	0.04 a	0.02 bc	0.06 b	1.2 c
%	16	24	117	25	32	16	87	81	95	18
AID	0.5 c	0.02 b	0.002 c	0.01 b	0.01 c	0.04 d	0.02 b	0.01 c	0.04 c	0.6 d
%	8	9	29	20	20	4	44	53	58	9
**Larnaka**										
BOD	13.9 a	0.13 a	0.01 c	0.12 a	0.09 a	3.7 a	0.15 a	0.01 c	0.13 b	18.2 a
AOD	4.9 b	0.10 b	0.06 a	0.03 b	0.05 b	1.8 b	0.10 b	0.04 a	0.30 a	7.4 b
%	35	77	516	30	62	49	64	411	227	40
AGD	2.4 c	0.04 c	0.03 b	0.01 b	0.03 c	0.5 c	0.10 b	0.02 b	0.10 c	3.2 c
%	17	35	234	10	33	13	58	258	68	17
AID	1.3 c	0.02 c	0.01 d	0.01 b	0.03 c	0.2 c	0.10 b	0.01 c	0.10 c	1.7 c
%	9	17	57	7	33	5	66	136	64	9
**Sirora**										
BOD	13.40 a	0.12 a	0.01 c	0.10 a	0.09 a	1.6 a	0.064 a	0.015 b	0.07 b	15.5 a
AOD	2.6 b	0.10 a	0.09 a	0.06 b	0.07 b	1.2 b	0.066 a	0.030 a	0.24 a	4.4 b
%	19	87	927	60	79	72	103	194	321	28
AGD	1.2 c	0.03 b	0.03 b	0.02 c	0.03 c	0.3 c	0.053 ab	0.012 bc	0.05 b	1.6 c
%	9	30	265	17	34	16	82	82	73	11
AID	0.8 c	0.02 b	0.01 c	0.02 c	0.03 c	0.1 c	0.050 b	0.010 c	0.04 b	1.0 c
%	6	18	108	15	32	6	77	63	56	7

Level of significance: Values with different letters denote significant difference as determined by a Tukey’s test (*p* ˂ 0.05). BOD: before oral digestion; AOD: after oral digestion; AGD: after gastric digestion; AID: after intestinal digestion. %: percentage remaining.

**Table 2:** Hydroxybenzoic acids group determined in pistachios before and after oral, gastric and intestinal in vitro digestion (nmol/g DW).

	3,4,5-Trihydroxybenzoic Acid	Benzene-1,2-diol	3-Galloyl-quinic Acid	3,4-Dihydroxy Benzoic Acid	Galloyl Shikimic Acid	3,4-Dihydroxy-5-((3,4,5-trihydroxybenzoyloxy) Benzoic Acid	Methyl Gallic Acid I	Methyl Gallic Acid II	Vanillic Acid Hexoside	Ellagic Acid	Benzoic Acid I	Benzoic Acid II	Benzoic Acid III	Total
**Aegina**														
BOD	0.25 ab	0.084 ab	0.003 a	13.19 a	0.013 a	0.0390 a	0.022 a	0.008 a	4.05 a	0.006 b	0.07 c	0.42 a	0.03 a	18.18 a
AOD	0.28 a	0.096 a	0.001 b	0.17 b	0.013 a	0.0040 b	0.004 b	0.004 b	3.19 a	0.013 a	0.31 a	0.18 b	0.01 b	4.28 b
%	111	115	19	1	99	9	19	55	79	203	470	43	40	24
AGD	0.29 a	0.069 b	0.001 b	0.17 b	0.011 a	0.0010 b	0.001 b	0.002 c	0.72 b	0.013 a	0.15 b	0.14 b	0.01 b	1.58 bc
%	116	83	22	1	85	2	5	21	18	212	226	33	49	9
AID	0.22 b	0.030 c	0.001 b	0.08 b	0.003 b	0.0010 b	0.001 b	0.001 c	0.34 b	0.014 a	0.09 c	0.11 b	0.01 b	0.90 c
%	88	36	25	1	20	1	4	14	8	214	148	25	46	5
**Golden Hills**														
BOD	0.098	0.032 a	0.005 a	4.53 a	0.004 a	0.0060 a	0.010 a	0.005 a	1.46 a	0.004 b	0.04 c	0.22 a	0.024 a	6.43 a
AOD	0.110	0.032 a	0.003 b	0.07 b	0.002 b	0.0004 b	0.002 b	0.001 b	0.89 b	0.013 a	0.14 a	0.06 b	0.007 c	1.33 b
%	112	101	50	2	44	6	18	28	61	339	390	26	28	21
AGD	0.126	0.025 ab	0.002 b	0.05 b	0.001 c	0.0002 b	0.001 b	0.001 c	0.19 c	0.013 a	0.08 b	0.05 b	0.008 b	0.55 b
%	128	80	44	1	30	4	7	15	13	340	227	22	34	9
AID	0.110	0.015 b	0.001 c	0.04 b	0.001 d	nd	0.001 b	0.001 c	0.13 c	0.013 a	0.09 b	0.04 b	0.008 b	0.45 b
%	112	48	11	1	13		5	12	9	347	252	20	32	7
**Kastel**														
BOD	0.13 ab	0.051 a	0.006 a	6.12 a	0.0042 a	0.0090 a	0.028 a	0.007 a	2.91 a	0.004 b	0.05 d	0.27 a	0.025 a	9.62 a
AOD	0.16 a	0.055 a	0.004 b	0.10 b	0.0021 b	0.0007 b	0.002 b	0.002 b	1.56 b	0.013 a	0.18 a	0.07 b	0.007 b	2.17 b
%	122	108	69	2	50	8	7	25	54	368	357	27	28	23
AGD	0.16 a	0.038 b	0.004 b	0.09 b	0.0017 c	0.0003 b	0.001 b	0.001 c	0.36 c	0.013 a	0.10 b	0.04 b	0.007 b	0.82 bc
%	116	75	71	2	40	3	2	12	12	368	205	16	29	8
AID	0.10 b	0.017 c	0.001 c	0.03 b	0.0004 d	0.0002 b	0.001 b	0.001 c	0.18 c	0.013 a	0.07 c	0.05 b	0.007 b	0.47 c
%	75	33	15	1	10	2	2	9	6	362	150	17	26	5
**Kerman**														
BOD	0.12 bc	0.019 c	0.008 a	2.74 a	0.0040 a	0.0104 a	0.015 a	0.006 a	2.38 a	0.004 b	0.05 d	0.16 a	0.024 a	5.53 a
AOD	0.16 a	0.028 a	0.006 b	0.05 b	0.0019 b	0.0005 b	0.001 b	0.001 b	1.90 b	0.013 a	0.21 a	0.05 b	0.007 c	2.43 b
%	139	151	71	2	43	5	10	23	80	321	463	34	31	44
AGD	0.15 ab	0.024 b	0.005 b	0.05 b	0.0019 b	0.0004 b	0.001 b	0.001 b	0.43 c	0.013 a	0.12 b	0.05 b	0.009 b	0.86 c
%	131	127	57	2	43	4	4	14	18	333	262	32	40	16
AID	0.09 c	0.015 d	0.001 c	0.02 b	0.0004 c	nd	0.001 b	0.001 c	0.18 c	0.013 a	0.09 c	0.04 b	0.007 c	0.45 c
%	75	78	11	1	9		3	12	7	320	199	24	29	8
**Larnaka**														
BOD	0.17 d	0.055 c	0.018 a	7.60 a	0.017 a	0.0363 a	0.035 a	0.009 a	5.53 a	0.004 c	0.08 d	0.29 a	0.025 a	13.86 a
AOD	0.52 a	0.094 a	0.015 a	0.28 b	0.019 a	0.0022 b	0.004 b	0.005 b	3.41 b	0.018 ab	0.34 a	0.14 b	0.010 c	4.86 b
%	314	17	82	4	110	6	13	50	62	441	454	48	38	35
AGD	0.46 b	0.068 b	0.014 a	0.25 b	0.017 a	0.0005 b	0.001 b	0.002 c	1.12 c	0.020 a	0.27 b	0.11 b	0.015 b	2.35 c
%	278	124	76	3	99	1	3	21	20	499	357	38	58	17
AID	0.25 c	0.036 d	0.003 b	0.09 b	0.004 b	0.0003 b	0.001 b	0.002 c	0.59 c	0.015 b	0.13 c	0.11 b	0.015 b	1.25 c
%	153	66	16	1	24	1	4	20	11	381	170	38	61	9
**Sirora**														
BOD	0.19 c	0.072 a	0.027 a	10.27 a	0.011 a	0.0394 a	0.076 a	0.012 a	2.46 a	0.005 b	0.05 c	0.19 a	0.024 a	13.42 a
AOD	0.31 a	0.079 a	0.030 a	0.16 b	0.011 a	0.0031 b	0.006 b	0.006 b	1.70 b	0.014 a	0.19 a	0.08 b	0.008 c	2.59 b
%	159	109	110	2	97	8	8	47	69	257	413	42	33	19
AGD	0.30 a	0.057 b	0.032 a	0.17 b	0.010 a	0.0009 b	0.002 b	0.003 c	0.34 c	0.015 a	0.17 a	0.05 b	0.011 b	1.15 c
%	154	79	117	2	90	2	2	23	14	275	369	25	43	9
AID	0.26 b	0.035 c	0.007 b	0.09 b	0.002 b	0.0003 b	0.001 b	0.001 c	0.19 c	0.016 a	0.08 b	0.06 b	0.009 b	0.76 c
%	135	49	27	1	21	1	1	11	8	289	177	33	39	6

Values with different letters are significantly different as determined by a Tukey’s test. BOD: before oral digestion; AOD: after oral digestion; AGD: after gastric digestion; AID: after intestinal digestion. %: percentage remaining.

**Table 3:** Flavan-3-ol group determined in pistachios before and after oral, gastric and intestinal in vitro digestion (nmol/g DW).

	Gallocatechin	Catechin	Epicatechin	Epigallo Catechin Gallate	Epi Catechin Gallate	Epiafzelechin3-Gallate	Afzelechin I	Afzelechin II	Afzelechin III	Total
**Aegina**										
BOD	0.326 a	0.271 a	0.037 a	2.41 a	0.052 a	0.008 a	0.004 a	0.004 a	0.006 a	3.12 a
AOD	0.293 ab	0.096 b	0.025 a	1.17 b	0.027 b	0.005 b	0.001 b	0.001 b	0.002 c	1.63 b
%	90	36	67	49	51	67	27	27	34	52
AGD	0.184 b	0.036 b	0.006 b	0.19 c	0.006 c	0.002 c	0.001 c	0.001 c	0.004 b	0.43 c
%	57	13	16	8	12	22	24	24	61	14
AID	0.036 c	0.013 b	0.005 b	0.06 c	0.004 c	0.002 c	0.001 c	0.001 c	0.003 b	0.13 c
%	11	5	13	3	7	20	24	23	49	4
**Golden Hills**										
BOD	0.056 a	0.142 a	0.075 a	0.23 a	0.038 a	0.010 a	0.004 a	0.004 a	0.006 a	0.57 a
AOD	0.051 a	0.053 b	0.032 b	0.14 b	0.022 b	0.006 b	0.001 b	0.001 b	0.002 c	0.31 b
%	91	38	44	60	57	57	25	26	27	54
AGD	0.026 b	0.017 c	0.008 c	0.02 c	0.005 c	0.002 c	0.001 c	0.001 c	0.003 b	0.09 c
%	46	12	10	11	13	19	24	24	41	16
AID	0.007 b	0.007 c	0.004 c	0.01 c	0.003 c	0.002 c	Nd	0.001 c	0.002 bc	0.04 c
%	12	5	5	4	8	16		24	37	6
**Kastel**										
BOD	0.084 a	0.251 a	0.127 a	0.56 a	0.079 a	0.016 a	0.004 a	0.004 a	0.007 a	1.13 a
AOD	0.062 b	0.078 b	0.056 b	0.32 b	0.047 b	0.009 b	0.001 b	0.001 b	0.002 c	0.58 b
%	73	31	44	58	59	59	26	25	27	52
AGD	0.030 c	0.019 c	0.007 c	0.04c	0.008 c	0.002 c	0.001 c	0.001 c	0.003 b	0.11 c
%	36	8	6	7	10	16	24	23	44	10
AID	0.003 d	0.005 c	0.004 c	0.02 c	0.006 c	0.002 c	0.001 c	0.001 c	0.002 c	0.04 c
%	4	2	3	3	7	13	24	23	31	4
**Kerman**										
BOD	0.120 a	0.291 a	0.177 a	0.36 a	0.055 a	0.016 a	0.004 a	0.0039 a	0.005 a	1.03 a
AOD	0.104 b	0.080 b	0.074 b	0.27 b	0.029 b	0.010 b	0.001 b	0.0010 b	0.001 c	0.57 b
%	87	27	42	75	52	63	25	25	29	55
AGD	0.052 c	0.031 c	0.013 c	0.05 c	0.008 c	0.003 c	0.001 c	0.0009 c	0.002 b	0.16 c
%	43	11	7	15	15	20	24	24	42	16
AID	0.006 d	0.007 c	0.005 c	0.01 d	0.003 c	0.002 c	0.001 c	0.0009 c	0.001 c	0.04 d
%	5	2	3	4	6	13	24	24	31	4
**Larnaka**										
BOD	0.398 a	0.625 a	0.120 a	2.44 a	0.090 a	0.012 a	0.004 a	0.0045 a	0.008 a	3.71 a
AOD	0.257 b	0.151 b	0.034 b	1.31 b	0.042 b	0.006 b	0.001 b	0.0012 b	0.005 c	1.81 b
%	65	24	28	54	47	54	29	28	56	49
AGD	0.181 c	0.070 c	0.008 b	0.20 c	0.009 c	0.002 c	0.001 c	0.0010 c	0.006 b	0.48 c
%	45	11	7	8	9	17	25	23	74	13
AID	0.040 d	0.027 d	0.007 b	0.09 c	0.007 c	0.002 c	0.001 c	0.0010 c	0.006 b	0.19 c
%	10	4	6	4	7	18	25	23	74	5
**Sirora**										
BOD	0.187 a	0.275 a	0.081 a	0.97 a	0.062 a	0.017 a	0.004 a	0.004 a	0.006 a	1.60 a
AOD	0.175 a	0.105 b	0.043 b	0.77 b	0.038 b	0.013 b	0.001 b	0.001 b	0.002 c	1.15 b
%	93	38	53	80	61	80	27	26	29	72
AGD	0.107 b	0.032 bc	0.006 c	0.09 c	0.006 c	0.003 c	0.001 c	0.001 c	0.002 bc	0.25 c
%	57	12	8	10	10	17	24	24	41	16
AID	0.027 c	0.013 c	0.005 c	0.04 c	0.003 c	0.002 c	0.001 c	0.001 c	0.003 b	0.09 c
%	14	5	6	4	6	14	23	23	50	6

Values with different letters are significantly different as determined by a Tukey’s test. BOD: before oral digestion; AOD: after oral digestion; AGD: after gastric digestion; AID: after intestinal digestion. %: percentage remaining.

**Table 4:** Catabolites produced during in vitro colonic fermentation in pistachios (nmol/g).

	3,4-DiOHPAA	3,4-DiOHPPA	4-OHBA	3-PAA	4OH3MeOHPPA	4OH3MeOHCA	3OH4MeOHCA	3-PPA	3-OHPAAC	4-OHPPA	3-OHPPA	3,4DiOHPVL	Total
	**Aegina**												
0 h	nd	nd	nd	nd	nd	nd	nd	nd	nd	nd	nd	nd	nd
4 h	nd	nd	nd	nd	0.38 d	nd	nd	nd	nd	nd	nd	nd	0.38 d
8 h	nd	nd	nd	nd	0.69 b	nd	nd	nd	nd	nd	nd	nd	0.69 d
24 h	0.15 ab	23.10 b	2.41	0.07 a	0.98 a	<LOQ	2.97 ab	3.89 a	20.19 cd	32.90 a	77.33	11.36 b	175.36 ab
	**Golden Hills**												
0 h	nd	nd	nd	nd	nd	nd	nd	nd	nd	nd	nd	nd	nd
4 h	nd	nd	nd	nd	0.37 c	nd	nd	nd	nd	nd	nd	nd	0.37 d
8 h	nd	nd	nd	nd	0.43 bc	nd	nd	nd	0.50 e	nd	nd	nd	0.93 d
24 h	0.10 b	14.57 cd	1.98	0.03 b	1.24 a	<LOQ	2.84 ab	2.57 b	55.90 ab	19.29 b	72.72	7.11 e	178.35 ab
	**Kastel**												
0 h	nd	nd	nd	nd	nd	nd	nd	nd	nd	nd	nd	nd	nd
4 h	nd	nd	nd	nd	0.44 bc	nd	nd	nd	nd	nd	nd	nd	0.44 d
8 h	nd	nd	nd	nd	0.58 bc	nd	nd	nd	nd	nd	nd	nd	0.58 d
24 h	0.08 b	12.64 d	1.88	0.03 b	0.99 ab	<LOQ	2.77 ab	2.84 b	42.53 bc	18.68 b	69.65	7.67 d	159.77 b
	**Kerman**												
0 h	nd	nd	nd	nd	nd	nd	nd	nd	nd	nd	nd	nd	nd
4 h	nd	nd	nd	nd	0.48 bc	nd	nd	nd	nd	nd	nd	nd	0.48 d
8 h	nd	nd	nd	nd	0.49 bc	nd	nd	0.22 c	0.46 e	nd	nd	nd	1.17 d
24 h	0.12 b	18.50 c	1.95	0.02 bc	1.18 a	<LOQ	2.68 ab	2.79 b	28.85 cd	15.33 b	74.66	9.08 c	156.203 b
	**Larnaka**												
0 h	nd	nd	nd	nd	nd	nd	nd	nd	nd	nd	nd	nd	nd
4 h	nd	nd	nd	nd	0.43 bc	nd	nd	nd	nd	nd	nd	nd	0.43 d
8 h	nd	nd	nd	nd	0.50 bc	nd	nd	nd	nd	nd	nd	nd	0.50 d
24 h	0.23 a	30.08 a	2.17	0.07 a	1.05 a	<LOQ	2.46 b	2.83 b	35.25 bc	34.39 a	75.36	17.37 a	201.76 ab
	**Sirora**												
0 h	nd	nd	nd	nd	nd	nd	nd	nd	nd	nd	nd	nd	nd
4 h	nd	nd	nd	nd	0.38 c	nd	nd	nd	nd	nd	nd	nd	0.38 d
8 h	nd	nd	nd	nd	0.36 c	nd	3.36 a	0.38 c	0.35 e	nd	nd	nd	4.45 c
24 h	0.13 b	14.73 cd	2.20	0.04 b	1.12 a	<LOQ	2.86 ab	2.42 b	70.53 a	41.76 a	70.05	9.36 c	215.20 a

a Average values., nd: not detected; ˂ LOQ: under the limit of quantification. Values with different letters are significantly different as determined by a Tukey’s test. 3,4-DiOHPAA: 3,4-Dihydroxyphenylacetic acid; 3,4-DiOHPPA: 3-(3′,4′-Dihydroxyphenyl)propanoic acid; 4-OHBA: 4-Hydroxybenzoic acid; 3-PAA: 3-Phenylacetic acid; 4OH,3MeOHPPA: 3-(4′-Hydroxy-3′-methoxyphenyl)propanoic acid; 4OH3MeOHCA: 4′-Hydroxy-3′-methoxycinnamic acid; 3OH4MeOHCA: 3′-Hydroxy-4′-methoxycinnamic acid; 3PPA: 3-(Phenyl)propanoic acid; 3OHPAAC: 3-Hydroxyphenylacetic acid; 4OHPPA: 3-(4′-Hydroxyphenyl)propanoic acid; 3OHPPA: 3-(3′-Hydroxyphenyl)propanoic acid; 3,4diOHPVL: 3,4-Dihydroxyphenyl-ɣ-valerolactone.

## Data Availability

No new data were created.

## Supplementary Materials

The following supporting information can be downloaded at: https://www.mdpi.com/article/10.3390/ijms24054975/s1.
