# Supplementary material for: Effect of In Vitro Gastrointestinal Digestion and Colonic Fermentation on the Stability of Polyphenols in Pistachio (Pistacia Vera L.)
Source: Int J Mol Sci. 2023 Mar 4;24(5):4975. doi: 10.3390/ijms24054975 (PMC10003603; doi:10.3390/ijms24054975)
Supplement: Supplementary file 1 [file ijms-24-04975-s001.zip › ijms-2241558-supplementary.pdf]

Table S1. UHPLC-HRMS characteristics of the polyphenols identified in pistachio varieties

RT (min)	Compound	Chemical Formula	[m/z]⁻ Theoretical	Error (ppm)	MSIMI level[a]
	Hydroxybenzoic acids				
2.88	3,4,5-Trihydroxybenzoic acid	$C_7H_6O_5$	169.0131	-0.59	1
3.15	Benzene-1,2-diol	$C_6H_5O_3$	125.0233	-1.60	1
3.38	Theogallin	$C_{14}H_{16}O_{10}$	343.0663	-0.87	2
6.6	Galloylshikimic acid	$C_{14}H_{14}O_9$	325.0554	3.38	2
8.69	3,4-Dihydroxy-5-((3,4,5-trihydroxybenzoyl)oxy)benzoic acid	$C_{14}H_{10}O_9$	321.0241	2.18	2
8.69	Methylgallic acid I	$C_8H_7O_5$	183.0287	1.64	2
11.72	Methylgallic acid II	$C_8H_7O_6$	183.0287	1.64	2
9.28	Vanillic acid	$C_8H_7O_4$	167.0339	1.80	1
9.87	Vanillic acid hexoside	$C_{14}H_{18}O_9$	329.0867	3.34	2
10.9	Ellagic acid	$C_{14}H_6O_8$	300.9978	4.32	1
12.31	Benzoic acid	$C_7H_5O_2$	121.0284	0.00	1
9.88	Benzoic acid I	$C_8H_7O_4$	167.0338	2.39	2
11.71	Benzoic acid II	$C_8H_7O_4$	167.0338	2.39	2
13.82	Benzoic acid III	$C_8H_7O_4$	167.0338	2.39	2
6.02	3,4-Dihydroxybenzoic acid	$C_7H_6O_4$	153.0182	-1.31	1
	Galloyl derivatives				
2.29	Mono-galloyl-glucose I	$C_{13}H_{16}O_{10}$	331.0659	3.02	2
6.25	Mono-galloyl-glucose II	$C_{13}H_{16}O_{10}$	331.0659	3.02	2
3.09	Galloyl dihexose	$C_{19}H_{26}O_{15}$	493.1188	0.20	2
8.39	Digalloyl hexose I	$C_{20}H_{20}O_{14}$	483.0769	2.28	2
8.92	Digalloyl hexose II	$C_{20}H_{20}O_{14}$	483.0769	2.28	2
8.94	Hydroxy-methoxyphenylgalloyl hexoside I	$C_{20}H_{22}O_{12}$	453.1027	2.43	2
9.63	Hydroxy-methoxyphenylgalloyl hexoside II	$C_{20}H_{22}O_{12}$	453.1027	2.43	2
9.89	Tetra-O-galloyl glucoside I	$C_{34}H_{28}O_{19}$	787.0994	1.40	2
10.65	Tetra-O-galloyl glucoside II	$C_{34}H_{28}O_{19}$	787.0994	1.40	2
11.4	Pentagalloylglucose	$C_{41}H_{32}O_{26}$	939.1098	0.64	2
	Hydroxycinnamic acids				
8.07	5-O-caffeoylquinic acid	$C_{16}H_{18}O_9$	353.0867	0.28	1
9.17	O-caffeoylquinic acid II	$C_{16}H_{18}O_9$	353.0867	0.28	2
11.37	Sinapic acid	$C_{11}H_{12}O5$	223.0600	2.24	1
8.93	Sinapic acid derivative	$C_{11}H_{12}O_5$	223.0600	2.24	2
10.61	4′-Hydroxycinnamic acid	$C_9H_8O_3$	163.0395	-2.45	1
8.51	4′-Hydroxy-3′-methoxycinnamic acid derivate	$C_{10}H_{10}O_4$	193.0495	1.55	2
7.67	3-(3′,4′-Dihydroxyphenyl)propanoic acid	$C_9H_9O_4$	181.0495	0.55	1

	Flavones				
8.54	Luteolin hexoside	$C_{21}H_{20}O_{11}$	447.0921	1.79	2
14.07	Luteolin	$C_{15}H_{10}O_{6}$	285.0393	3.51	1
11.01	Apigenin hexoside	$C_{21}H_{20}O_{10}$	431.0972	1.16	2
	Flavonols				
10.74	Quercetin 3-rutinoside	$C_{27}H_{30}O_{16}$	609.145	0.98	1
10.18	Myricetin hexoside	$C_{21}H_{20}O_{13}$	479.082	1.67	2
12.48	Myricetin	$C_{15}H_{10}O_{8}$	317.0291	3.47	1
10.55	Quercetin hexoside I	$C_{21}H_{20}O_{12}$	463.0871	1.73	2
10.98	Quercetin hexoside II	$C_{21}H_{20}O_{12}$	463.0871	1.73	2
10.60	Quercetin galloyl glucoside I	$C_{28}H_{24}O_{16}$	615.098	1.46	2
11.43	Quercetin galloyl glucoside II	$C_{28}H_{24}O_{16}$	615.098	1.46	2
14.32	Quercetin	$C_{15}H_{10}O_{7}$	301.0342	3.65	1
	Flavan-3-ols				
6.33	Gallocatechin	$C_{15}H_{14}O_{7}$	305.0655	3.61	2
8.61	Catechin	$C_{15}H_{14}O_{6}$	289.0707	4.15	1
9.54	Epicatechin	$C_{15}H_{14}O_{6}$	289.0707	2.42	1
9.72	Epigallocatechin gallate	$C_{22}H_{18}O_{11}$	457.0765	0.44	1
11.05	Epicatechin gallate	$C_{22}H_{18}O_{10}$	441.0816	0.91	1
11.88	Epiafzelechin 3-gallate	$C_{22}H_{18}O_{9}$	425.0867	1.88	2
12.42	Afzelechin I	$C_{15}H_{14}O_{5}$	273.0757	2.93	2
12.94	Afzelechin II	$C_{15}H_{14}O_{5}$	273.0757	2.93	2
15.23	Afzelechin III	$C_{15}H_{14}O_{5}$	273.0757	2.93	2
	Flavanones				
11.02	Eriodictyol hexoside	$C_{21}H_{22}O_{11}$	449.1078	1.11	2
13.88	Eriodictyol	$C_{15}H_{12}O_{6}$	287.055	2.79	2
11.98	Naringenin hexoside	$C_{21}H_{22}O_{10}$	433.1129	1.39	2
11.98	Naringenin	$C_{15}H_{12}O_{5}$	271.0601	2.21	1
	Flavanonols				
11.39	Taxifolin	$C_{15}H_{12}O_{7}$	303.0499	3.30	1
	Stilbenes				
10.87	Resveratrol hexoside	$C_{20}H_{22}O_{8}$	389.1231	2.31	2

[a] Annotation from Summer et al. Compounds identified at MSIMI level 1 means there was a commercially available standard. Compounds identified at MSIMSI level 2 were tentatively identified. RT: retention time.

Table S2. UHPLC-HRMS Characteristics of Phenolic Acid Catabolites Identified in Pistachio and its by-products Faecal Incubates

Rt (min)	Catabolites	Chemical Formula	$[m/z]^-$ Theoretical	Error (ppm)	MSIMI level[a]
7.32	3,4-Dihydroxyphenylacetic acid	$C_8H_7O_4$	167.0338	-2.99	1
8.18	4-Hydroxybenzoic acid	$C_7H_5O_3$	137.0233	0.00	1
8.84	3-(3′,4′-Dihydroxyphenyl)propanoic acid	$C_9H_9O_4$	181.0495	0.55	1
8.85	3-(Phenyl)acetic acid	$C_8H_7O_2$	135.0440	0.74	1
10.12	3-Hydroxyphenylacetic acid	$C_9H_9O_3$	151.0389	0.66	2
10.28	3,4-Dihydroxyphenyl-ɣ-valerolactone	$C_{11}H_{12}O_4$	207.0657	0.415	2
10.91	3-(3-methoxy-4-hydroxyphenyl)propionic acid	$C_{10}H_{11}O_4$	195.0652	3.59	1
10.95	3-(4′-Hydroxyphenyl)propanoic acid	$C_9H_9O_3$	165.0546	0.66	1
11.45	4′-Hydroxy-3′-methoxycinnamic acid	$C_{10}H_{10}O_4$	193.0495	1.04	1
11.6	3′-Hydroxy-4′-methoxycinnamic acid	$C_{10}H_90_4$	193.0495	2.59	1
11.74	3-(3′-Hydroxyphenyl)propanoic acid	$C_7H_6O_4$	165.0546	0.66	1
14.45	3-(Phenyl)propionic acid	$C_9H_9O_2$	149.0597	0.67	1

[a] Annotation from the Summer et al. Compounds identified at MSIMI level 1 means there was a commercially available standard. RT: retention time.

Table S3. Hydroxybenzoic acids group in pistachios during fermentation (nmol/ g)

	3,4,5-Trihydroxybenzoic acid	Benzene-1,2-diol)	3-Galloyl-quinic acid	3,4-Dihydroxy benzoic acid	Galloyl shikimic acid	Vanillic acid hexoside	Ellagic acid	Benzoic acid derivative II	Total
					**Aegina**				
**0 h**	0.21 bc	nd	nd	0.154 ab	0.0005	0.57	0.244 a	0.198 a	1.38 a
**4 h**	0.45 a	nd	nd	0.166 a	nd	nd	0.237 b	0.176 b	1.03 b
**8 h**	0.34 ab	nd	nd	0.148 ab	nd	nd	0.236 b	0.169 b	0.90 c
**24 h**	0.03 c	0.07	nd	0.039 c	nd	nd	nd	nd	0.14 d
					**Golden Hills**				
**0 h**	<LOQ	nd	nd	0.046 b	0.0004	0.55	0.243 a	0.13	0.97 a
**4 h**	0.06 a	nd	nd	0.071 a	nd	nd	0.237 b	0.13	0.50 b
**8 h**	0.05 a	nd	nd	0.060 ab	nd	nd	0.242 ab	0.13	0.49 c
**24 h**	<LOQ	0.05	nd	0.024 c	nd	nd	nd	nd	0.07 d
					**Kastel**				
**0 h**	0.31 a	nd	0.005	0.092 a	0.0004	0.601	0.246	0.174 a	1.43 a
**4 h**	0.05 c	nd	nd	0.078 a	nd	nd	0.239	0.138 b	0.51 c
**8 h**	0.11 b	nd	nd	0.082 a	nd	nd	0.241	0.145 ab	0.58 b
**24 h**	<LOQ	0.04	nd	0.017 b	nd	nd	nd	nd	0.06 d
					**Kerman**				
**0 h**	<LOQ	nd	0.004	0.036 b	0.0004	0.553	0.238	0.136 b	0.97 a
**4 h**	0.09 b	nd	nd	0.059 a	nd	nd	0.243	0.149 a	0.54 c
**8 h**	0.14 a	nd	nd	0.060 a	nd	nd	0.240	0.158 a	0.59 b
**24 h**	<LOQ	0.06	nd	0.015 c	nd	nd	nd	nd	0.07 d
					**Larnaka**				
**0 h**	0.26 a	nd	0.01	0.173 a	0.001	0.609	0.238	0.249 a	1.54 a
**4 h**	0.37 a	nd	nd	0.204 a	nd	nd	0.235	0.175 b	0.98 b
**8 h**	0.35 a	nd	nd	0.180 a	nd	nd	0.238	0.185 b	0.95 c
**24 h**	0.04 b	0.11	nd	0.031 b	nd	nd	nd	nd	0.18 d
					**Sirora**				
**0 h**	0.06 b	nd	0.01	0.113 b	0.0005	0.544	0.236	0.144	1.10 a
**4 h**	0.12 b	nd	nd	0.140 a	nd	nd	0.239	0.137	0.64 c
**8 h**	0.29 a	nd	nd	0.167 a	nd	nd	0.235	0.145	0.84 b
**24 h**	<LOQ	0.04	nd	0.038 c	nd	nd	nd	nd	0.08 d

Values with different letters are significantly different as determined by Tukey test.

Table S4. Flavan-3-ols group in pistachios during fermentation (nmol/ g)

	Catechin	Epi catechin	Epigallo catechin gallate	Epi catechin gallate	Epiafzelechin3 -gallate	Afzelechin III	Total
				**Aegina**			
**0 h**	0.018 a	0.022 a	nd	0.013	0.013	0.019 a	0.08 c
**4 h**	0.010 b	0.020 ab	0.13	0.013	0.012	0.018 b	0.21 a
**8 h**	0.002 c	0.020 ab	0.13	0.012	0.012	0.018 b	0.20 b
**24 h**	<LOQ	0.019 b	nd	0.012	nd	nd	0.03 d
				**Golden Hills**			
**0 h**	<LOQ	0.019	nd	0.013	0.013	0.018	0.06 b
**4 h**	<LOQ	0.018	0.11	0.012	0.012	0.018	0.18 a
**8 h**	<LOQ	0.019	nd	0.012	0.009	0.018	0.06 c
**24 h**	<LOQ	0.019	nd	0.012	nd	nd	0.03 d
				**Kastel**			
**0 h**	0.022	0.026 a	nd	0.014 a	0.014 a	0.020 a	0.10 b
**4 h**	<LOQ	0.018 b	nd	0.012 b	0.012 b	0.018 b	0.06 c
**8 h**	<LOQ	0.020 ab	0.12	0.012 b	0.012 b	0.018 b	0.19 a
**24 h**	<LOQ	0.020 ab	nd	0.012 b	nd	nd	0.03 d
				**Kerman**			
**0 h**	0.002 a	0.021 a	nd	0.012 a	0.013	0.018	0.07 b
**4 h**	0.001 b	0.022 a	nd	0.012 a	0.013	0.019	0.07 a
**8 h**	<LOQ	0.021 a	nd	0.012 a	0.012	0.018	0.06 c
**24 h**	<LOQ	0.018 b	nd	0.011 b	nd	nd	0.03 d
				**Larnaka**			
**0 h**	0.057 a	0.025 a	nd	0.014 a	0.013	0.021 a	0.13 b
**4 h**	0.025 b	0.022 b	0.12	0.012 b	0.012	0.018 b	0.21 a
**8 h**	0.021 b	0.023 b	0.12	0.012 b	0.012	0.018 b	0.21 a
**24 h**	<LOQ	0.018 c	nd	0.012 b	nd	nd	0.03 c
				**Sirora**			
**0 h**	0.007	0.020 a	0.12	0.013	0.013	0.018	0.19 a
**4 h**	<LOQ	0.019 ab	0.12	0.012	0.012	0.018	0.18 b
**8 h**	<LOQ	0.020 a	0.12	0.012	0.012	0.018	0.18 b
**24 h**	<LOQ	0.018 b	nd	0.012	nd	nd	0.03 c

Values with different letters are significantly different as determined by Tukey test.
